# Oral Rehabilitation for a Patient with Cocaine-Induced Midline Destructive Lesions

**DOI:** 10.1155/2024/7109261

**Published:** 2024-06-19

**Authors:** Antoine Berberi, Elie Azar

**Affiliations:** ^1^Faculty of Dental Medicine, Lebanese University, Beirut, Lebanon; ^2^Department of ENT, Faculty of Medical Sciences, Lebanese University, Beirut, Lebanon

## Abstract

**Background:**

Cocaine is the second most consumed drug worldwide, more than 0.4% of the global population, and has become a real public health problem in recent years. Its inhalation causes significant centrofacial lesions, grouped under the name cocaine-induced midline destructive lesion (CIMDL). These destructions are due to the conjunction of the vasoconstrictor, local prothrombogenic effects, and cytotoxic effects of cocaine. The ischemia produced by this substance is due to vasoconstriction that leads to nasal tissue necrosis and perforation of the nasal septum secondary to chondral necrosis. *Case Presentation*. A 36-year-old man, previously grappling with cocaine addiction, was hospitalized to undergo comprehensive clinical, microbiological, and radiological examinations because he was suffering from the emergence of crusts and ulceration in the nasal mucosa, accompanied by a palate perforation, a 39°C fever, and chills. Standard bacteriological culture was positive for coagulase-negative staphylococci and *Escherichia coli*, while mycological culture was positive for *Candida tropicalis*. The CT scan images of the sinuses confirmed the presence of palatal perforation and total destruction of the nasal septum, cartilaginous portion, maxillary sinus medial wall, lower and middle turbinates, and middle meatus. Nasal endoscopy revealed an exposition of the bony wall and displayed the exposition of the occipital bone's clivus. A diagnosis of CIMDL was confirmed. Antibiotic therapy was decided based on antibiogram results by the consulting microbiologist. Debridement of necrotic tissue was done by nasal endoscopy with local cleaning and was repetitive during the first week to maintain the best cleanliness possible. The patient was discharged with oro-nasal hygiene instructions and referred for prosthetic rehabilation. As for the cocaine addiction, the patient was in follow-up with a psychologist in a specialized centre.

**Conclusion:**

The care is multidisciplinary. Psychological help and assistance are essential to guide patients to become cocaine free and to avoid a relapse. Weaning is a prerequisite for surgery. Rehabilitation of speech and swallowing is necessary. Many local flaps or micro-anastomoses are possible.

## 1. Introduction

Cocaine is the second most consumed drug worldwide, which has become a real public health problem in recent years, more than 0.4% of the global population [1].

In USA, 2.0% (over 18 years) were current illegal cocaine users [1]. In Europe, the consumption varies between 1.2% and 2.2% with an age range between 15 and 64 years [2].

Cocaine can be administered orally, intranasally, intravenously, or smoked as a rock [1, 2].

When cocaine is used intranasally, individuals inhale cocaine powder through their nostrils, where the sinosal mucosa is most commonly affected due to the high concentration of absorption through the nasal tissue bloodstream [3, 4].

Chronic cocaine abuse produces various effects, which can be systemic or local [5].

The local effects are due, fundamentally, to the ischemia produced by this substance due to vasoconstriction and to the chemical irritation of the epithelium of the upper airway produced by the added adulterants, which lead to atrophic changes in the nasal mucus [6].

If consumption is prolonged, the ischemic process prevents the correct healing of the lesions, leading to tissue necrosis and perforation [7]. One of the local effects due to chronic inhalation of this substance is perforation of the nasal septum secondary to chondral necrosis [8, 9].

Recent studies show that extensive destructive midline lesions induced by cocaine can appear up to 5% of cocaine-consuming patients; they are relatively rare but serious entities [8–15].

The adverse effects of “snorting” cocaine may lead to serious orofacial lesions such as nasal septum perforation and soft and hard palate destruction, gingival ulcerations, and tooth enamel erosion, in addition to alterations of the feel of odor and chronic sinusitis that could provoke ischemic osseocartilaginous necrosis and lead to damage to midline structures [13–15]. Those reported lesions are named cocaine-induced midline destructive lesions (CIMDLs) [9–12].

We report a case of a 36-year-old man, a cocaine addict, suffering from CIMDL, referred for treatment and prosthetic management.

## 2. Observation

A 36-year-old man, previously grappling with cocaine addiction, was referred for prosthetic rehabilitation due to oro-nasal-antral communication issues.

The narrative unfolded a month ago, marked by the emergence of crusts and ulceration in the nasal mucosa, accompanied by a palate perforation, a 39°C fever, and chills.

Functionally, the patient experienced significant nasal pain, a sense of obstruction, distress during the passage of food and liquids from the mouth into the nose, swallowing difficulties, and frequent epistaxis resulting from the wide palatal perforation.

Upon consultation with a physician, the patient was hospitalized to undergo comprehensive clinical, microbiological, and radiological examinations.

**Figure 1:**
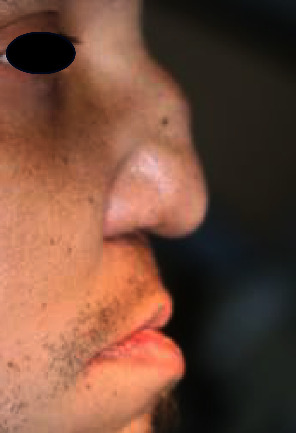
Typical saddle-nose deformities with columellar retraction and nasal collapse.

**Figure 2:**
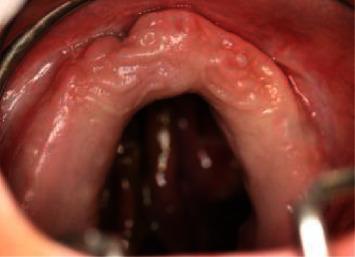
Complete edentulous maxilla with an oro-nasal-antral communication.

The patient presented with a saddle nose, and a clinical oral examination unveiled a broad perforation in the palatal bone, coupled with a fully edentulous maxilla (Figures 1 and 2).

The blood count showed the presence of polymorphonuclear neutrophils at 80.3% and lymphocytes at 12.9% of total leukocytes. A biological inflammatory syndrome was evident (CRP 31.6 mg/L). Creatinine was 1.3 mg/dL, without proteinuria. Liver tests were normal, with SGPT at 6 U/L and SGOT at 9 U/L. The serology test for HIV was negative.

Standard bacteriological culture was positive for coagulase-negative staphylococci and *Escherichia coli*, while mycological culture was positive for *Candida tropicalis*.

Antibiograms revealed that cefadroxil was active against *E. coli* and coagulase-negative staphylococci and amphotericin B was active against *Candida tropicalis*.

**Figure 3:**
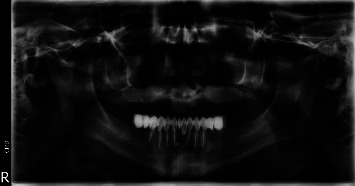
The panoramic X-ray reveals a complete edentulous maxilla with a radiolucent image of the nasal cavity and maxillary sinuses showing nasal destruction with sinus communication.

Radiological exams of the lungs were normal, with no significant cervical lymphadenopathy. The panoramic X-ray revealed a completely edentulous maxilla with a radiolucent image of the nasal cavity and maxillary sinuses, indicating nasal destruction with sinus communication (Figure 3).

**Figure 4:**
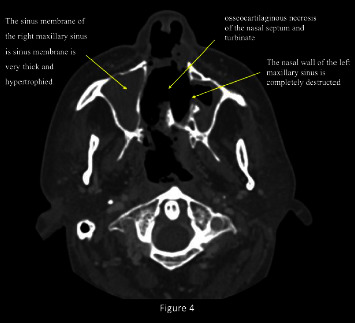
Axial CT scan image showing the necrosis of the osseo-cartilagineous of the nasal septum and turbinate, and the destruction of the nasal wall of the left maxillary sinus and the sinus membrane of the right maxillary membrane is very thick and hypertrophied.

The axial images of the CT scan of the sinuses confirmed the presence of palatal perforation and total destruction of the nasal septum, cartilaginous portion, maxillary sinus medial wall, lower and middle turbinates, and middle meatus (Figure 4).

**Figure 5:**
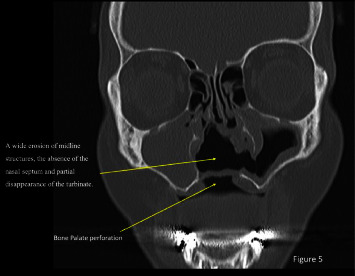
Coronal section of the CT scan revealed a wide erosion of midline structures, the absence of the nasal septum, and partial disappearance of the turbinates. Note the palatal perforation that establishes an oronasal communication.

Coronal CT scan images showed a total obstruction of the right maxillary sinus associated with a complete destruction of bulla ethmoidalis and ethmoid cells (Figure 5).

Additionally, they revealed that the nasolacrimal duct, superior turbinate, frontal and sphenoid sinuses, and sella turcica were undamaged.

**Figure 6:**
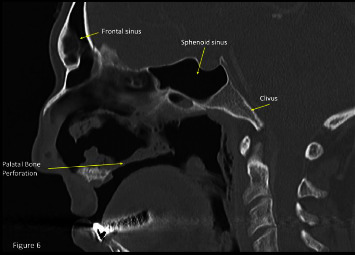
The sagittal images of the CT scan revealed bony and cartilaginous destruction of the nasal septum and the palate. The rhinopharyngeal mucosa is eroded with occipital bone clivus exposition.

The sagittal images of the CT scan revealed bony and cartilaginous destruction of the nasal septum and palate. The rhinopharyngeal mucosa is eroded, with occipital bone clivus exposition (Figure 6).

The ear, nose, and throat (ENT) evaluation revealed signs of rhinopharyngeal inflammation and confirmed the existence of a palatal perforation, situated in the middle and posterior part of the hard palate and bordered by a marginal necrosis of the soft tissues.

Nasal endoscopy revealed an explosion of the bony wall and displayed the exposition of the occipital bone's clivus. A diagnosis of CIMDL was finally confirmed.

Antibiotic therapy was decided based on antibiogram results by the consulting microbiologist. The patient received intravenously broad-spectrum antibiotic based on metronidazole 500 mg four times a day, cefadroxil 2 g three times a day, and amphotericin B 50 mg four times a day for 10 days. The patient's health improvement was observed for 3 to 4 days after the beginning of the treatment, and inflammation signs decreased one week later and CRP levels moved from 31.6 mg/L to 4.3 mg/L.

Debridement of necrotic tissue was done by nasal endoscopy with local cleaning and was repetitive during the first week to maintain the best cleanliness possible.

The patient was discharged with the needed instructions for a good oral and nasal hygiene.

As for the cocaine addiction, the patient was in follow-up with a psychologist in a specialized centre.

The plan was to provide the patient with a complete removable prosthesis with a hollow bulb obturator to achieve defect obturation.

The prosthetic rehabilitation began with a primary maxillary impression using an irreversible hydrocolloid. The final impression, involving a personalized resin tray with the obturator cast framework, was crafted using putty and light-addition silicon (Elite HD+; Zhermack, Italy). Subsequently, the obturator prosthesis was created using the conventional technique.

**Figure 7:**
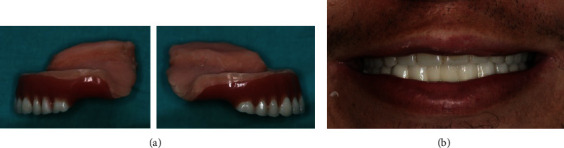
(a) Lateral views of the finished and polished prosthesis obturator. (b) Prosthesis obturator inserted intraorally.

The hollow bulb obturator was fashioned using acrylic resin (Probase Cold; Ivoclar Vivadent, Schaan, Liechtenstein), underwent finishing and polishing, and was then inserted intraorally (Figures 7(a) and 7(b)).

The patient received instructions for oral hygiene procedures, and regular monthly appointments were scheduled to assess oral mucosa healing and prevent infections.

## 3. Discussion

The precise prevalence of CIMDL lesions among cocaine users remains unknown, though some authors report a frequency of septal perforations among users of up to 4.8% [16].

Clinical examination reveals crust and nasal ulcerations, as well as more or less extensive perforation of the septum [15]. The most severe forms may be accompanied by an extension of necrosis to the middle and upper turbinates, as well as perforation of the soft and/or hard palates and nasal deformation [8–16].

Smith et al. reported that the CIMDL diagnosis is based on the presence of two lesions from the following criteria: soft or and hard palate perforation, destruction of the lateral and inferior walls of the nose and choanae, and paranasal sinus necrosis, leading to the breakdown of the nose [11].

General signs as fever, asthenia, and arthralgia are rare or mild. Their presence should prompt consideration of a systemic process. These forms are not accompanied by ophthalmological damage (except for infectious complications such as orbital cellulitis) or hearing issues [5].

The presence of an inflammatory syndrome is rare and should be a priority when investigating local superinfection of the lesions or other infectious complications in these high-risk areas (e.g., endocarditis, hepatitis, and skin infection) [5, 9–13].

Clinical symptoms include nasal discharge and regurgitation, disabling centrofacial pain, and collapse of the nasal pyramid due to the destruction of bone and cartilaginous structures [13–15].

The nose appears flat, and alveolar and labial involvement is exceptional [11, 13].

Intraoral examination reveals varying degrees of palatal necrosis [8]. Involvement of the tear ducts and the orbit is not uncommon [11].

The intensity of destruction lesions varies and can include the mucosa or extend deeper into the septum, causing devastation of the turbinate and walls of the nose, and it may also implicate the external area of the philtrum and columella [9–16].

When the perforation of the soft and/or hard palate occurs, patients may suffer from hypernasal speech and oronasal reflux, resulting in a significant undesirable impact on social life [8, 11, 13].

Several classifications of CIMDL have been proposed, with the first one described by Westreich and Lawson [16], based on the location of the necrotizing lesion. More recently, Nitro et al. [17] suggested a CIMDL classification based on the extension of the destructive lesions in the sinonasal area, categorized into four grades:

- Grade 1: nasal septum region, representing 99.2% of CIMDL patients.
- Grade 2a: inferior turbinate and maxillary sinus medial wall regions, representing 59% of CIMDL patients.
- Grade 2b: palatal region, representing 29.9% of CIMDL patients.
- Grade 3: ethmoid bone, middle turbinate, and superior turbinate regions, representing 22.8% of CIMDL patients.
- Grade 4: papyracea, orbit, or skull base regions, representing 7.9% of CIMDL patients.

Nasal inhalation of cocaine triggers compression of blood vessels, and addiction extends the constriction of the nasal lining blood vessels and leads to the death of these tissues [7, 18].

Local and general vasoconstriction, increased myocardial oxygen requirements, and the prothrombotic state create a state of hypoxia which promotes ischemia [18].

Local superinfections are common. An increase in the consumption of cocaine intended for analgesic purposes maintains and increases necrotic phenomena that can lead to perforation of the septum and loss of cartilage and eventual deformity of the nose known as “cocaine nose” [19, 20].

In the differential diagnosis of destructive processes of the centrofacial midline in young patients, continued cocaine abuse should be included as a frequent etiological possibility [6, 9, 11].

Among the differential diagnoses, granulomatosis with polyangiitis (GPA) constitutes a particular trap. Many conditions other than GPA can mimic CIMDL [19–24].

However, it must be remembered that septal perforation can also be due to other causes such as trauma, rheumatic diseases (Wegener's granulomatosis), neoplasms (lymphoma and squamous-cell carcinoma), and chronic infections (mycobacteria, leishmaniasis, actinomycosis, mucormycosis, tuberculosis, and tertiary syphilis in particular) or inflammatory conditions (eosinophilic granulomatosis with polyangiitis, sarcoidosis, and associated diseases) [19–24].

Trauma (mechanical, chemical, burns, and microtrauma) must be eliminated [9].

Long-term local corticosteroid therapy, whether associated with vasoconstrictors, may be responsible for comparable lesions [25].

The treatment is complex and must include both medical-surgical and psychological management, since before carrying out any action, it is essential to abandon the consumption of this substance [26].

Debridement of necrotic tissue, local dressings, and saline washing may prove useful, along with the use of antibiotics in patients with infectious processes such as sinusitis or rhinitis [26–28].

It is recommended to delay surgical repair of bone plate defects until after a period of habituation, and during this period, abstinence is monitored through urinary values of a cocaine metabolite (benzoylecgonine) and, in the case of nasopalatine fistula, palatal prostheses can be used to prevent nasal regurgitation [27, 28].

Obturator prostheses can serve as an interim solution before surgical treatment.

While they enhance speech and swallowing, their adaptation can be challenging [28].

Our patient presented extensive necrosis of the nasal cavity with total destruction of most nasal and sinus structures, resulting in the resorption of the palate bone and a wide perforation of the soft tissue, categorized as Grade 3 according to Nitro et al.

Bacterial and mycological infections contributed to the necrosis and destruction.

Antibiotherapy coupled with mycological therapy, along with local debridement by nasal endoscopy, facilitated the patient's recovery. The maxillary obturator will significantly improve his quality of life. Ongoing psychological support is crucial to assist and guide him towards achieving freedom from cocaine use before surgical planning can be proposed.

## 4. Conclusion

Diagnosing CIMDL is a complex process due to the similarities it shares with other diseases. Psychologists play a crucial role in assessment and ongoing support to achieve complete independence from cocaine.

The management of CIMDL necessitates a multidisciplinary approach involving collaboration between medical and dental professionals for comprehensive treatment, prosthetic rehabilitation, and surgical reconstruction.

## Data Availability

All data are included within the article.
